# Potentials of Non-Invasive ^18^F-FDG PET/CT in Immunotherapy Prediction for Non–Small Cell Lung Cancer

**DOI:** 10.3389/fgene.2021.810011

**Published:** 2022-02-04

**Authors:** Xuhe Liao, Meng Liu, Rongfu Wang, Jianhua Zhang

**Affiliations:** Department of Nuclear Medicine, Peking University First Hospital, Beijing, China

**Keywords:** PET/CT, FDG, immunotherapy, immune checkpoint inhibitors (ICI), lung cancer, NSCLC, response, prognosis

## Abstract

The immune checkpoint inhibitors (ICIs), by targeting cytotoxic-T-lymphocyte-associated protein 4, programmed cell death 1 (PD-1), or PD-ligand 1, have dramatically changed the natural history of several cancers, including non–small cell lung cancer (NSCLC). There are unusual response manifestations (such as pseudo-progression, hyper-progression, and immune-related adverse events) observed in patients with ICIs because of the unique mechanisms of these agents. These specific situations challenge response and prognostic assessment to ICIs challenging. This review demonstrates how ^18^F-FDG PET/CT can help identify these unusual response patterns in a non-invasive and effective way. Then, a series of semi-quantitative parameters derived from ^18^F-FDG PET/CT are introduced. These indexes have been recognized as the non-invasive biomarkers to predicting the efficacy of ICIs and survival of NSCLC patients according to the latest clinical studies. Moreover, the current situation regarding the functional criteria based on ^18^F-FDG PET/CT for immunotherapeutic response assessment is presented and analyzed. Although the criteria based on ^18^F-FDG PET/CT proposed some resolutions to overcome limitations of morphologic criteria in the assessment of tumor response to ICIs, further researches should be performed to validate and improve these assessing systems. Then, the last part in this review displays the present status and a perspective of novel specific PET probes targeting key molecules relevant to immunotherapy in prediction and response assessment.

## 1 Introduction

Immune checkpoint inhibitors (ICIs), by targeting cytotoxic-T-lymphocyte-associated protein 4 (CTLA-4), programmed cell death 1 (PD-1), or PD-ligand 1 (PD-L1), have yielded durable anti-tumor responses and long-term remissions in non–small cell lung cancer (NSCLC) and other cancer types. Consequently, the clinical importance and use of ICIs have skyrocketed.

ICIs reactivate the patient’s immune system through T lymphocytes against tumor cells. This mechanism differs from that of cytotoxic or targeted agents, which could result in the development of inflammation at the tumor sites and subsequent anti-tumor responses (Missailidis, 2008). These responses are of unique patterns, pseudo-progression, hyper-progression (HPD), long duration of response, and disease regression, which continue after treatment discontinuation, especially pseudo-progression and HPD, which can influence the treatment evaluation in many clinical cases and predict the entirely reverse prognosis. Moreover, such an increased immune system activity can activate T immune cells in a variety of normal tissues, causing autoimmune side effects, also known as immune-related adverse events (irAEs).

Therefore, the novel manifestations, previously seldom seen during conventional therapies and frequently described under immunotherapy, have challenged responsive and prognostic assessment to ICIs. Nevertheless, a substantial number of patients still do not derive benefit from checkpoint inhibitors. Clinical data showed that the responding rate of ICIs is closely correlated with biomarker expression in tumor specimens, for example, PD-L1 status (Borghaei et al., 2015; Herbst et al., 2016; Reck et al., 2016), tumor mutational burden (TMB) (Galvano et al., 2021), microsatellite instability-high (MSI-H) (Gregg et al., 2019; Zhao et al., 2019), and mismatch repair-deficient (MMR-deficient) (Gregg et al., 2019; Zhao et al., 2019). In particular, PD-L1 expression and TMB have been recognized as the valuable predictive factors for NSCLC patients with ICIs: a recent meta-analysis demonstrated that NSCLC patients with high PD-L1 expression did benefit the most from a single-agent ICI treatment in the first-line setting (Passiglia et al., 2021), and another meta-analysis resulted in a proven benefit in overall survival (OS) in favor of ICIs in the TMB-high NSCLC population (Galvano et al., 2021). MSI-H and MMR-deficient are proposed as the predictors for anti-PD-1/anti-PD-L1 immunotherapy efficacy in colorectal cancer (Zhao et al., 2019). Given that MSI-H lung cancers are extremely rare, MSI testing/MMR immunohistochemistry (IHC) is not considered as routine in lung cancer closely associated with high expression of PD-1/PD-L1 and shows durable responses to PD-1 blockade (Gregg et al., 2019). However, the heterogeneity within tumors limits the effectiveness of predictive value of these biomarkers based on IHC and next-generation sequencing (NGS) of tumor specimens. Additionally, since not all advanced NSCLC patients are suitable for tumor biopsy, biopsy specimens are not always readily available for the pathological assay. Thus, non-invasive biomarkers are urgently needed for the prediction of potential responsiveness of ICIs.

2-Deoxy-2-(^18^F)-fluoro-D-glucose (^18^F-FDG) positron emission tomography/computed tomography (PET/CT) takes advantage of non-invasively evaluating the glucose metabolic level to determine the tumor burden all over the body. Parameters derived from ^18^F-FDG PET/CT, relevant to glucose metabolism on the molecular level, are demonstrated to be correlated with the treatment response and survival, which shows a potential capability of recognizing and tracing biomarkers related to responsiveness and prognosis. Therefore, ^18^F-FDG PET/CT has been widely used to determine the tumor stage and drug efficacy including the responsiveness of ICIs in NSCLC patients (Na et al., 2017; Liao et al., 2020).

Meanwhile, new positron molecular probes targeting key molecules associated with immunotherapy have been developed and would be used to improve the assessing specificity and effectiveness for NSCLC patients treated with immunotherapy.

Thus, this review summarizes the potential applications of ^18^F-FDG PET/CT as a non-invasive method for the prediction and tracking of responsiveness and prognosis of ICIs in NSCLC patients. A perspective of novel PET molecular probes in immunotherapy prediction and response assessment is also discussed.

## 2 Manuscript

### 2.1 ^18^F-FDG PET/CT

#### 2.1.1 Identification of Unusual Response Patterns

The mechanism of ICIs leads to unique manifestations during and after immunotherapy. Pseudo-progression and HPD belong to the unusual response patterns, which occur more frequently in ICIs than in other treatments (chemotherapy, target treatment, angiogenic inhibitors, etc.) (Iacovelli et al., 2015; Mellema et al., 2015; Barrón et al., 2018; Ferrara et al., 2018; Zhou et al., 2020).

Immune system-related pseudo-progression was first described in melanoma patients receiving ipilimumab (an anti-CTLA-4 antibody) in 2008. Pseudo-progression is described as a process with an increase in tumor size or metabolic tumor burden or the appearance of new lesions before the occurrence of a subsequent decrease or stability (Costa et al., 2021). Among patients with advanced NSCLC, the rate of pseudo-progression is 3–5% (Gettinger et al., 2015; Ferrara et al., 2018; Fujimoto et al., 2019), which is lower than that among patients with melanoma, for whom the equivalent rate was reported as up to 10% (Hodi et al., 2008). Besides the primary manifestations above (varying tumor size and metabolic tumor burden, emergence of new lesions), other rare forms of pseudo-progression include pleural or pericardial effusion (Kanazu et al., 2018; Song et al., 2019), intestinal perforation (Kim et al., 2019), and brain pseudo-progression (Melian et al., 2018). The certain mechanisms of pseudo-progression are unknown. There are some hypotheses trying to explain this pattern, including delayed and weaker activation of the immune system, immune cell infiltration, intracellular and vasogenic edema, and inflammation and intra-tumor hemorrhage (Fujimoto et al., 2019) (Gerwing et al., 2019). The chronic activation of immune system results in delay control of tumor growth, which can be shown as the elevation of FDG uptake in tumor sites with or without increase in tumor size and appearance of new lesions during the early period after administration of ICI agents. In contrast, immune cell infiltration and activation by checkpoint blockade antibodies influence the tumor immune microenvironment and then might promote the glycolysis of cancer cells, leading to the increase in FDG uptake at the early treatment phase (Tomita et al., 2018; Tomita et al., 2020). In terms of the diverse hypotheses for pseudo-progression, the mechanisms of glucose-metabolic elevation are completely different, but the FDG-uptake increase and the subsequent decrease or stability indeed can be observed through multiple PET/CT scans to help distinguish pseudo-progression from other responses. It is reported that pseudo-progression could occur between weeks 4 and 20 from baseline (Evangelista et al., 2019; Costa et al., 2021) and this time interval from baseline to pseudo-progression tends to be shorter than that from baseline to response. Additionally, patients with pseudo-progression would experience a markedly superior survival to those with normal response situations (Kurra et al., 2016; Solinas et al., 2017; Zhou et al., 2020). Thus, imaging reassessment is suggested after 4–8 weeks (never more than 12 weeks) (Costa et al., 2021).

HPD was depicted as a process of expansive growth or change in the rate of tumor progression that is grossly different from baseline, causing a detrimental effect on the patients. It is another atypical response pattern of ICIs and becomes a big challenge in clinical practices because of the higher incidence and worse prognosis. HPD’s molecular mechanisms are still unclear. Champiat et al. concluded the following probable explanations: 1) expansion of regulatory T cells; 2) exhaustion of compensatory T cells; 3) modulation of protumorigenic immune cell subsets; 4) oncogenic pathway activation; and 5) activation of aberrant inflammation (Champiat et al., 2018). The expansive growth and proliferation of tumors inevitably induce the raising of tumoral glucose consumption leading to the rapid and sustained increase in FDG uptake. The incidence of HPD in NSCLC patients is about 5–19.2% (Ferrara et al., 2018), and HPD could be apparently associated with a poor prognosis (Ferrara et al., 2018). Multi-center retrospective research with 406 advanced NSCLC patients demonstrated patients experiencing HPD treated with ICIs had significantly lower overall survival (OS) compared with patients with normal progressive disease (median OS, 3.4 months [95% confidence interval (CI), 2.8–7.5 months] *vs.* 6.2 months (95% CI, 5.3–7.9 months); hazard ratio, 2.18 [95% CI, 1.29–3.69]; *P* = 0.003). Nevertheless, it is difficult to differentiate HPD from other response patterns within 8 weeks of starting treatments and the second early imaging evaluation is proposed after this period (more than 8 weeks after immunotherapy) to properly adjust therapeutic programs (Costa et al., 2021).

In fact, there is no consensus on the definitions for pseudo-progression and HPD, and their molecular mechanisms are still unclear. The range of their incidences is quite wide, which reflects the heterogeneity of cancer subtypes and therapies (type of agents and dose).

**FIGURE 1:**
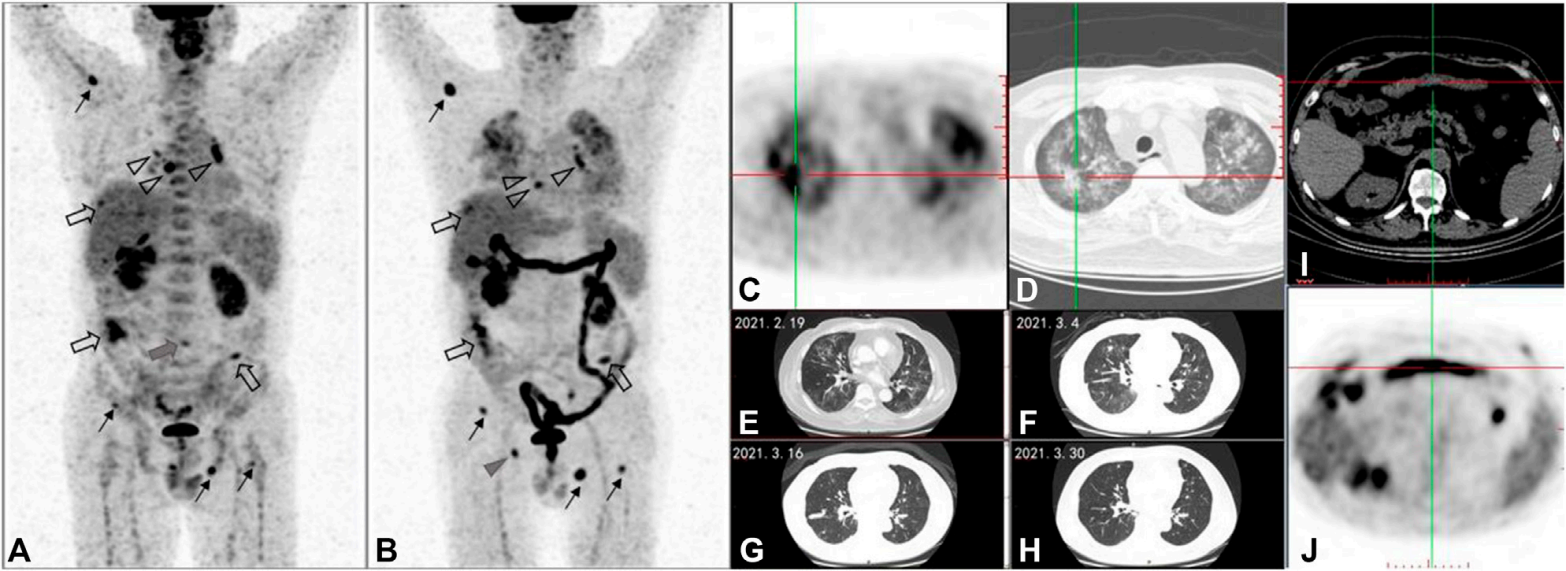
A 58-year-old male patient with metastatic intrahepatic cholangiocarcinoma was treated with the third line of ipilimumab. After four cycles of treatment, compared with ^18^F-FDG PET/CT maximum intensity projection (MIP) image **(A)** before treatment, the posttreatment ^18^F-FDG PET/CT **(B-D)**, **(I,J)** demonstrates the following: 1) multiple soft tissue density nodules in the peritoneum, which were larger in volume and higher in FDG uptake than before (**(A,B)** hollow arrows); 2) multiple nodules without FDG uptake in both lungs, some of which are larger in volume than before and some of which have no change compared with before (cannot be shown in MIP images- **(A,B)**); 3) the lymph nodes with increased FDG uptake in mediastinal areas and bilateral hilar, which were smaller in volume and FDG-uptake lower than before (**(A,B)** hollow triangles); and 4) multiple foci of increased FDG uptake in bones: the FDG-uptake degree in vertebral body of lumbar 4 was significantly lower than before (**(A)** gray triangle); the right acetabular lesion was the new lesion (**(B)** gray triangle); the remaining ostial lesions were higher than those before (**(A,B)** black line arrows). According to PERCIMT criteron: SMD was confirmed for the number of the newly FDG-positive lesions is less than 4. Meanwhile, in the light of imPERCIST5, PMD was also evaluated for the sum of SUL_peak_ of the patient’s top five target lesions after treatment was more than 30% higher than that of the top five target lesions before treatment. As a result of clinical follow-up, the patient was confirmed PD and those lesion above were validated as metastases. PD was determined for the appearance of new lesion based on PECRIT. PMD was determined for new FDG-avid lesion at SCAN-2, which can be considered as UPMD in the line with iPERCIST. But the patient didn’t receive the next ^18^F-FDG PET/CT between days 21 and 28 after treatment or 4–8 weeks later, so it could not be evaluated according to PECRIT and iPERCIST. Follow-up results showed the PFS for ICI was 6 months. Additionally, this posttreatment PET/CT displayed: 1) newly patchy ground glass density foci in both lungs, with increased FDG uptake (C- PET axial image, D-CT axial image); 2) diffuse increased FDG uptake in ascending colon, transverse colon, descending colon, sigmoid colon and rectum (i-PET axial image) which is new compared with the previous one, and the corresponding colonic walls were not significantly thickened (**(J)**-CT axial image). The patchy ground glass shadows of both lungs gradually disappeared during chest CT follow-up (**(E–H)**-CT axial images). Combined with clinical information, the patchy ground glass shadows of both lungs were diagnosed as immunerelated pneumonia. Under the administration of MDT, the diffuse increase of glucose metabolism in the colon was considered as immune-related colitis.

Reactivation of the immune system through the use of ICIs has led to the development of a new series of side effects named irAEs. The precise pathophysiology underlying irAEs is not clearly understood. Due to the whole immunity enhancement, irAEs can potentially occur in any system of the whole body and can vary in involved systems, incidence, and severity of grade according to each particular agent and its dose and tumor types. irAEs commonly manifest within the first 3 months of administration for most drugs and can also occur at any time during treatment, even after discontinuation of ICI therapy (Costa et al., 2021). These effects could be mistaken as the disease progression, including HPD resulting in inappropriate strategies. Although most irAEs are mild and can be managed through transient immunosuppression with corticosteroids, high-grade events often require hospitalization and specialized treatment because some of them are life-threatening. In addition, multiple studies reported that patients who experienced irAEs demonstrate marked improvements in progression-free survival (PFS), OS, and overall response rate compared to those lacking toxicity (Das and Johnson, 2019). Therefore, early and accurate identification of irAEs is essential to aid in the diagnosis and management of patients and to reduce associated morbidity. Some effects can be apparent on ^18^F-FDG PET/CT imaging. A correct interpretation of ^18^F-FDG PET/CT images could be complicated for imaging manifestations of the unusual patterns and adverse reactions. However, the whole-body scan is also essential to discover these situations and know their characteristic localizations, especially when some effects are not always associated with the presence of clinical symptoms and signs. Thus, PET imaging with ^18^F-FDG can ensure close clinical monitoring and medical intervention when necessary to avoid serious complications. In some situations, imaging features from ^18^F-FDG PET/CT can effectively improve the diagnosis of particular effects. Taking the intestinal adverse effect for example, after carefully considering and ruling out other possibilities such as inflammatory bowel disease or continuing metformin before undergoing PET/CT, diffuse or segmental FDG uptake in bowel with or without thickening wall could be recognized as the toxicity of ICIs (Figure 1). Additionally, multiple scans (before, during, and after treatment) based on assessment criteria (see 2.1.3) can further contribute to distinguishing among these situations. Table 1 displays the common irAEs, including the features in PET/CT to help to recognize these effects.

**TABLE 1:** Common immune-related adverse events.

irAEs	Incidence in NSCLC	Symptoms, signs, and laboratory test	Susceptibility factors	Imaging features	Intervention
Gastrointestinal toxicity	Anti-PD-1: 8% (Davies and Duffield, 2017)	Diarrhea, abdominal pain, vomiting, fever, and hematochezia (Sosa et al., 2018)		Diffuse or segmental bowel wall thickening, with increased enhancement and FDG uptake (Costa et al., 2021)	Symptomatic treatment, including oral hydration, oral or intravenous corticosteroids, and immunosuppressive agents; discontinuation of ICIs in severe cases (Sosa et al., 2018)
Dermatologic toxicity	Anti-PD-1: 9% (Davies and Duffield, 2017; Sosa et al., 2018)	Rash, pruritus, vitiligo, photosensitivity reactions, and xerosis cutis (Sosa et al., 2018)		Rarely shown at CT, MR, and PET/CT (Costa et al., 2021)	Symptomatic treatment, including topical corticosteroids and oral antihistamines (Sosa et al., 2018)
Endocrine toxicity					
Thyroiditis	Anti-PD-1: hyperthyroidism 1–8%, hypothyroidism 4–9% (Davies and Duffield, 2017)	Asymptomatic or with symptoms and signs of short-term hyperthyroidism and subsequent temporary or permeant hypothyroidism (Sosa et al., 2018)	Female, more cycles of ICIs (Davies and Duffield, 2017)	Normal or diffuse FDG uptake (Costa et al., 2021)	Monitor thyroid function monthly or every two cycles during ICI; temporary or persistent hormone replacement therapy, but the ICI may be continued (Sosa et al., 2018)
	Anti-PD-L1: hyperthyroidism 1%, hypothyroidism 4% (Davies and Duffield, 2017)				
Hypophysitis	Anti-PD-1: ≤ 1% (Sosa et al., 2018)	Fatigue, headache, and visual field changes; the abnormality of relevant hormone (Sznol et al., 2017)		Normal or diffuse FDG and uptake with or without the swollen size (Sznol et al., 2017)	Hormone replacement therapy, but the ICI may be continued (Sosa et al., 2018)
Adrenalitis	Rare (only case report) (Sznol et al., 2017)	Fatigue, postural dizziness, orthostatic hypotension, anorexia, weight loss, and abdominal discomfort; the abnormality of relevant hormone (Sznol et al., 2017)		Bilateral mild and diffuse gland enlargement with FDG uptake (Sznol et al., 2017)	Stress-dose and emergency corticosteroid administration when PAI is confirmed; long-term glucocorticoid and mineralocorticoid replacement (Sznol et al., 2017)
Hepatotoxicity	Anti-PD-1: 2% (Davies and Duffield, 2017)	Asymptomatic increase of ALT, AST, or total bilirubin (Sosa et al., 2018)	patients with prior autoimmune disease (Sosa et al., 2018)	Hepatomegaly, periportal edema, or heterogeneous liver enhancement, with or without diffuse FDG uptake (Costa et al., 2021)	Monitor transaminases and bilirubin twice a week during ICI. Oral corticosteroids when LFTs remain elevated after 1-2 weeks and re-starting the ICI once LFTs have improved and steroid has been tapered (Sosa et al., 2018)
Pancreatitis	4% (Sosa et al., 2018)	Elevated levels of pancreatic enzymes with or without abdominal pain, nausea, and vomiting (Sosa et al., 2018)		Diffuse pancreatic enlargement with FDG uptake at PET with or without peripancreatic fat stranding (Costa et al., 2021).	Routine monitoring of amylase and lipase is not recommended; symptomatic mild elevations of these enzymes should not be treated (Sosa et al., 2018)
Pulmonary toxicity	Anti-PD-1: 3–6%; anti-PD-L1: 4% (Davies and Duffield, 2017)	Highly variable in extent of severity (Sosa et al., 2018)	Male former smokers, previous lung radiation therapy, lung fibrosis (Davies and Duffield, 2017)	1. COP: multifocal GGOs and consolidations with a predominantly peripheral distribution; 2. NSIP: mild GGOs with a tendency for peripheral distribution; 3. HP: diffuse mild GGOs and centrilobular nodules; 4. AIP/ARDS: diffuse GGOs, consolidations, and lung volume loss	Systemic treatment with corticosteroids and antibiotics and withholding ICI treatment with 2–4 grades (Sosa et al., 2018)
		Asymptomatic or dry cough, fever, chest pain, progressive dyspnea, and fine inspiratory crackles (Sosa et al., 2018)	Such pulmonary opacities can show different intensities of FDG uptake with or without mediastinal lymphadenopathy and pleural effusions (Costa et al., 2021)		
Nephrotoxicity	Anti-PD-1: 1–3% (Davies and Duffield, 2017)	Asymptomatic elevation of creatinine (Sosa et al., 2018)		Rarely shown at CT, MR, and PET/CT.	Stopping any concomitant nephrotoxic drugs; evaluating etiologies; corticosteroid treatment and withholding ICI (Sosa et al., 2018)
Neurological toxicity	Anti-PD-1: < 1% (Davies and Duffield, 2017)	Polyneuropathy, facial paralysis, optic neuritis, GBS, myasthenia gravis, transverse myelitis, encephalitis, and aseptic meningitis (Sosa et al., 2018)		Imaging examinations, especially MR to rule out metastasis (Sosa et al., 2018)	Steroid treatment; higher doses or other procedures (e.g., intravenous immunoglobulin for GBS) might be required for more severe toxicity (Sosa et al., 2018)
Cardiotoxicity	Myocarditis 0.27% of anti-CTLA-4, 0.06% of anti-PD-1 (Sosa et al., 2018)	Heart failure, cardiomyopathy, heart block, myocardial fibrosis, myocarditis, pericarditis, cardiomyopathy, and arrhythmias (Sosa et al., 2018)		Abnormal signal could be detected by MR.	Consultation with cardiologists and treatment with steroids (Sosa et al., 2018)
Musculoskeletal and rheumatologic toxicity	Arthralgia: 43%, myalgia: 21%, arthritis/tenosynovitis: 1–7%, myositis/fasciitis: <1% (Sosa et al., 2018)	Arthralgia, myalgia, arthritis/tenosynovitis, myositis/fasciitis, rheumatoid arthritis, polymyalgia rheumatica, lupus erythematosus, and Sjögren syndrome (Sosa et al., 2018)		Joint effusion, synovial thickening, or tendon/muscle edema with increased enhancement and FDG uptake (Costa et al., 2021)	Symptomatic or low-dose steroids (Sosa et al., 2018)
Sarcoidosis or sarcoid-like reaction	5–7% (Nishino et al., 2018)	Asymptomatic (Nishino et al., 2018)		FDG avid enlarged lymph nodes of mediastinum, bilateral hilar, neck and abdomen; seldom manifestation: perilymphatic pulmonary nodules, focal lung consolidation, splenic or hepatic nodule (Nishino et al., 2018)	Holding ICIs without any specific treatment (Kelly et al., 2021)

irAEs: immune-related adverse events; CTLA-4: cytotoxic T-lymphocyte-associated protein 4; PD-1: programmed cell death 1; PD-L1: PD-ligand 1; CT: computed tomography; MR: magnetic resonance; PET/CT: positron emission tomography/computed tomography; ICIs: immune checkpoint inhibitors; FDG: fluorodeoxyglucose; PAI: primary adrenal insufficiency; ALT: alanine aminotransferase; AST: aspartate aminotransferase; LFTs: liver function tests; COP: cryptogenic organizing pneumonia; GGOs, ground-glass opacities; NSIP: nonspecific interstitial pneumonia; HP: hypersensitivity pneumonitis; AIP/ARDS: acute interstitial pneumonia/acute respiratory distress syndrome; GBS: Guillain–Barre syndrome.

The identification of pseudo-progression, HPD, and irAEs challenges the immunotherapy in NSCLC. According to the demonstrations above, during the early period of immunotherapy, the alterations of FDG uptake based on pseudo-progression and HPD may be similar. However, their mechanisms of the FDG uptake are entirely different. Additionally, some irAEs could further confound the diagnosis. Taking the sarcoidosis or sarcoid-like reaction, for example, these FDG avid enlarged lymph nodes may be misdiagnosed as lymph node metastases. Thus, the finds in ^18^F-FDG PET/CT imaging need to be compared with those of the subsequent PET/CT scans to confirm the kind of response (in 2.1.3, the assessment criteria based on ^18^F-FDG PET/CT displayed how to use PET/CT scans to confirm the response status). The quantitative parameters based on ^18^F-FDG PET/CT could help in differentiation (the role of parameters from ^18^F-FDG PET/CT in differentiation is shown in 2.1.2). As mentioned above, the recommended imaging assessment time differs among pseudo-progression, HPD, and irAEs. There is no uniform recommendation optimum time yet when those responses or events could be differentiated well. Thus, the complexity of these responses and events leads to the discernment process requiring a comprehensive analysis combined with multiple information such as clinical symptoms, signs, laboratory examinations, and imaging manifestations, which can be well implemented by the model of the multidisciplinary team (MDT) including nuclear medicine physicians.

#### 2.1.2 Parameters in Prognostic Evaluation for Immunotherapy

Generally, there are three imaging assessing approaches based on ^18^F-FDG PET/CT: visual interpretation and estimation of relative uptake, assessment of uptake over a defined time using semi-quantitative methods, and assessment of uptake from the time of injection to a defined endpoint using kinetic analysis (Shankar et al., 2006). Visual assessment is of subjectivity, and the results could vary among readers. Additionally, FDG accumulation is influenced by uptake time, blood glucose concentration, and partial-volume effects (Shankar et al., 2006). The visual comparison among different scans is unsuitable and less rigorous for evaluation. Full kinetic quantitative analysis can provide an absolute rate for FDG metabolism, which is independent of imaging time and contributes to the investigation of various components of glucose metabolism, such as transport and phosphorylation. Nevertheless, because of the complexity of such an approach, including patient compliance issues and the requirement for arterial blood sampling or dynamic imaging of a blood-pool structure to obtain a precise input function, the kinetic analysis has been used infrequently for treatment evaluation of malignancy (Weber et al., 1999).

The semi-quantitative method is a relatively objective approach to evaluate the metabolic tumoral status. Semi-quantitative parameters—standardized uptake value (SUV)—have been used to determine tracer uptake in attenuation-corrected PET images in ^18^F-FDG PET/CT, mainly representing the extent of glucose metabolism. However, it was reported that SUV based on ^18^F-FDG PET/CT is associated with molecules relevant to some signaling pathways, for instance, the mTOR pathway. Previous studies have shown SUV of FDG is significantly associated with PD-L1 expression (Takada et al., 2017), and the activation of the protein kinase B (AKT)–mTOR pathway increases PD-L1 protein expression in NSCLC (Kaira et al., 2014). Then SUV from ^18^F-FDG PET/CT could be relevant to the prognosis of immunotherapy in NSCLC.

The maximum SUV(SUV_max_) is widely used in clinical practices because of its high repeatability. However, SUV_max_, only representing the highest metabolic point in the volume of interest (VOI) determination, ignores the intra-tumoral and inter-tumoral heterogeneity. Other semi-quantitative parameters such as mean SUV (SUV_mean_), peak SUV(SUV_peak_), metabolic tumor volume (MTV), and total lesion glycolysis (TLG) can reflect more information on biological behaviors such as heterogeneity of tumors, therapeutic responsiveness, and prognosis. SUV_mean_ was defined as the average SUV related to the tumor burden. SUV_peak_ was the highest average SUV calculated within a spherical VOI (usually 1-cm-diameter region). MTV represents the metabolically active portion of the tumor, obtained through several SUV-based segmentation techniques. TLG is the product of SUV_mean_ x MTV. MTV and TLG, which consider volumetric information and reflect the whole-body tumor burden, have been more frequently proposed as prognostic factors for immunotherapy in NSCLC patients (Seban et al., 2020a; Seban et al., 2020b; Castello et al., 2020; Seban et al., 2020c; Polverari et al., 2020; Monaco et al., 2021). The latest relevant studies on ^18^F-FDG PET-derived parameters for predicting the response and prognosis of ICIs in NSCLC patients in the recent 3 years are displayed in Table 2. These research works demonstrated the value of baseline semi-quantitative parameters of ^18^F-FDG PET/CT in the responsiveness and prognosis of NSCLC patients treated with ICIs. However, under the semi-quantitative method, FDG accumulation is still affected by many technical, physiological, and pathological factors. Thus, the imaging-based comparative response evaluations during treatment for immune-treatment have been adopted.

**TABLE 2:** The studies on the predictive value of semi-quantitative parameters for immunotherapy in NSCLC patients.

Studies	Sample	ICIs	Outcomes	SUV	Design	Results
Monaco et al., 2021 (Monaco et al., 2021)	92 NSCLC	Nivolumab, pembrolizumab, or atezolizumab	1. Response	wbMTV, wbTLG, SUV_max_, SUV_mean_	Retrospective study	1. Patients who achieved disease control (CR, PR, SD) had significantly lower wb**MTV** median values than patients with PD (77 *vs*. 160.2, *p* = 0.039)
			2. OS			2. Patients with lower wb**MTV** and wb**TLG** had improved OS compared to patients with higher MTV (*p* = 0.03) and TLG (*p* = 0.05)
Seban et al., 2020 (Seban et al., 2020a)	63 advanced NSCLC with a PD-L1 TBS ≥50% 63	pembrolizumab	1. LTB	SUV_max_, SUV_mean_ wbMTV wbTLG	Multi-center retrospective study	1. In multivariate analyses, high wb**MTV** (>84 cm^3^) and high tumor **SUV**_**mean**_ (>10.1) remained independent factors for predicting LTB (OR 0.2; *p* = 0.03 and OR 3.7; *p* = 0.04) and PFS (HR 2.2; *p* = 0.02 and HR 0.5; *p* = 0.045)
			2. PFS			2. High **wbMTV** was significantly associated with poor OS (HR 3.1; *p* = 0.03)
			3. OS			
Polverariet al, 2020 (Polverari et al., 2020)	57 advanced NCSLC	Pembrolizumab or nivolumab or atezolizumab	1. PFS	MTV, TLG, SUV_max_	Retrospective study	1. **MTV** (*p* = 0.028) and **TLG** (*p* = 0.035) were significantly associated with progressive *vs*. non-progressive disease status (*p* = 0.035)
			2. OS			2. **TLG** had a higher probability of failing immunotherapy
			3. Response			
Chardin D et al., 2020 (Chardin et al., 2020)	75 NSCLC	Pembrolizumab or nivolumab	1. OS	SUV_max_, SUV_peak_, MTV, TLG	Prospective study	1. A high **MTV** and a high **TLG** were significantly associated with a lower OS (*p* < 0.001)
			2. ETD			2. **MTV** and **TLG** could reliably predict ETD (area under the ROC curve = 0.76, 95% CI: 0.65 to 0.87 and 0.72, 95% CI: 0.62 to 0.84, respectively)
Seban R et al., 2020 (Seban et al., 2020b)	63 advanced NSCLC with a PD-L1 TBS ≥50%	Pembrolizumab	1. PFS	wbMTV	Multi-center retrospective study	Patients have been grouped based on score combining the wb**MTV** and dNLR into the good, intermediate and poor
			2. OS			1. Median OS was 17.9 months (14.6 not reached) for the good group *vs.* 13.8 (95%CI 8.4–18.9) and 6.6 (CI 2.0–11.2) months for the intermediate and poor groups, respectively
			3. DCR			2. Median PFS was 15.1 (95%CI 12.1–20.0) months for the good group *vs.* 5.2 (1.9–8.5) and 1.9 (95%CI 1.3–2.5) months for the intermediate and poor groups, respectively
			4. ORR			3. The poor prognosis group was associated with DCR and ORR (*p* < 0.05)
Seban R D et al., 2020 (Seban et al., 2020c)	80 advanced NSCLC	Pembrolizumab or nivolumab or atezolizumab	1. PFS	Highest SUV_max_ of all lesions, wbMTV	Retrospective study	wb**MTV** > 75 cm^3^ combined with dNLR >3 were associated with shorter OS (HR 2.5, 95%CI 1.3–4.7 and HR 3.3, 95%CI 1.6–6.4) and absence of DCB (OR 0.3, 95%CI 0.1–0.9 and OR 0.4, 95%CI 0.2–0.9)
			2. OS			
			3. DCB classification			
Evangelista et al., 2019 (Evangelista et al., 2019)	32 metastatic NSCLC	Nivolumab	Response	wbSUV_max_ wbMTV, wbTLG	Retrospective study	wb**SUV**_**max**_ was significantly higher in patients without a response than those with a response to immunotherapy (median: 48.97 *vs*. 20.85; Student’s *t*-test: *p* = 0.002)

Bold values indicates the statistically significant indicators of studies. NSCLC: non–small cell cancer; ICIs: immune checkpoint inhibitors; SUV: standardized uptake value; OS: overall survival; wbMTV: whole-body metabolic tumor volume; wbTLG: whole-body total lesion glycolysis; SUV_max_: maximum SUV; SUV_mean_: mean SUV; CR: complete response; PR: partial response; SD: stable disease; PD-L1: programmed cell death ligand 1; LTB: long-term benefit; PFS: progression-free survival; OR: odds ratio; HR: hazard ratio; MTV: metabolic tumor volume; TLG: total lesion glycolysis; ETD: early treatment discontinuation; 95%CI: confidence interval; ROC: receiver operating characteristic; TBS: tumor proportion score; DCR: disease control rate; ORR: overall response rate; dNLR: neutrophils to lymphocytes ratio; DCB: disease clinical benefit; wbSUV_max_: whole-body SUV_max_.

It should be noted that SUL_peak_ is consistently employed as the only semi-quantitative parameter in functional criteria (see 2.1.3). SUV can be normalized to body mass, lean body mass, or body surface area. SUV normalized to lean body mass is named SUL. SUV normalized to body mass is the commonest parameter in clinical practices but cannot consider the relatively lower ^18^F-FDG accumulation in fatty tissues (Wahl et al., 1992) and are more dependent on body habitus across populations than SUL and SUV corrected for body surface area (Evangelista et al., 2020). Moreover, normalization to body surface area or lean body mass potentially reduces the effect of weight loss (which may occur during therapy) on subsequent SUV determinations. In addition, lean body mass may be the better method because of the availability of sex-specific corrections (Sugawara et al., 1999). Peak standardized uptake values normalized by lean body mass (SUL_peak_) were the highest average SUL calculated within a spherical VOI in the site of the most metabolically active tumor manifestation and of high repeatability. Therefore, SUL_peak_ has been used as an effective index for evaluating malignancies treatment, including immunotherapy.

#### 2.1.3 Metabolic-Based Response Assessment Criteria

Imaging-based response assessment is essential for the clinical management of immunotherapy. The role of the assessment system is as follows: 1) identifying non-responders; 2) confirming the response patterns; and 3) detecting irAEs.

Morphologic criteria are widely adopted in clinical trials and practices. These anatomic/structural assessing systems determine therapeutic effectiveness on the ground of the change of target-lesion size and the presence of new lesions principally. However, these anatomic response criteria cannot timely discern response without lesion volumetric changes such as cavitation, cystic change, intra-tumoral hemorrhage, and reduction in vascularity, which might occur after the use of immunotherapeutic agents and are considered physiopathologic features of a specific response to them (Costa et al., 2021). For example, as quite extensively used anatomic response criteria, Response Evaluation Criteria in Solid Tumors version 1.0 or version 1.1 (RECIST 1.0 or RECIST 1.1) define tumor responses into a series of four bins of response [complete response (CR), partial response (PR), stable disease (SD), and progressive disease (PD)]. Nevertheless, some tumor sizes shrink slowly, but patients live for a long period with SD evaluated by RECIST 1.0/1.1, so this stable-disease response could have highly beneficial outcomes (Benjamin et al., 2007; Llovet et al., 2008; Van den Abbeele, 2008). Thus, with such reductionism through RECIST, the potentially valuable information that may be important is lost (Wahl et al., 2009). An additional consideration for RECIST is that the response evaluation from the baseline and follow-up studies is based on a precise estimate, which requires repeatability and accuracy of the assessment. However, more misclassifications and variance in response are seen when a different reader assesses the baseline and follow-up studies (Erasmus et al., 2003; Wahl et al., 2009).

FDG uptake of malignant tissues depends on the tumor’s functional status, such as rate of glycolysis, expression of glucose transport, level of proliferation, and association with particular molecules. Thus, ^18^F-FDG is a marker of tumoral activity. In terms of the functional volume, metabolic extent, and whole-body burden of tumor, functional criteria with ^18^F-FDG PET/CT to assess immunotherapy responses have been adapted for lung cancer and melanoma patients. There are at least four FDG PET/CT criteria (Table 4):

1) Early Prediction of Response to Immune Checkpoint Inhibitor therapy (PECRIT) (Cho et al., 2017): PECRIT is the combining functional and anatomic assessing system based on the RECIST 1.1 and PET Response Criteria in Solid Tumors (PERCIST), which requires two PET/CT scans (first scan: before therapy; second scan: between days 21 and 28 on therapy). According to PECRIT, initial SD, defined by RECIST 1.1, should be assessed by ^18^F-FDG PET/CT (SCAN 2) after 3–4 weeks. On SCAN 2, when the percent change in SUL_peak_ per PERCIST criteria is more than 15.5%, the stable metabolic disease (SMD) will be confirmed, or when the percent change in SUL_peak_ is less than or equal to 15.5%, this pattern will be confirmed as progressive metabolic disease (PMD). As shown in Table 3, PECRIT admits the initial increase in FDG uptake during the early immunotherapeutic period and could effectively distinguish pseudo-progression and real progression by the particular threshold value of SUL_peak_ percent change (15.5%). Moreover, this evaluation criteria combined with morphologic and functional criteria to effectively reduce assessment cost. However, measurement of all tumor lesions, as suggested by this PECRIT, is not only time-consuming but also sometimes impossible, especially in patients with advanced disease from an aggressive malignancy, which may be associated with a high number of metastatic lesions and/or diffuse organ infiltration. In addition, although data from PECRIT original research demonstrated that sensitivity, specificity, and accuracy of this algorithm to predict response at 4 months after initiation of therapy were 100, 93.3, and 95.0%, respectively, PECRIT is just established through the study based on 20 advanced melanoma patients. Therefore the threshold value of SUL_peak_ could be further validated or statistically calculated based on large samples with multiple cancer types.

**TABLE 3:** ^18^F-FDG PET/CT assessing criteria for immunotherapy.

Criteria	CR/CMR	*p*R/PMR	SD/SMD	*p*D/PMD	Notes
PECRITCho et al., 2017 (Cho et al., 2017)	Disappearance of all target and non-target lesions without any new lesions. Any pathological lymph nodes must have a reduction in short axis to <10 mm. Determined by two observations not less than 4 weeks apart	At least a 30% decrease in the sum of maximum diameters of target lesions; no new lesions; no progression of disease	1. Does not meet the criteria for CR, PR, or PD, taking the smallest sum of the maximum diameters of target lesions as referencesAnd	1. Sum of the maximum diameter of lesions increased by >20% over the smallest achieved sum of maximum diameter. The appearance of one or more new lesions is always considered progressionOr	
			2. Then, ^18^F-FDG PET (SCAN 2) assessment is required after 3-4 weeks: i) when percent change in SULpeak per PERCIST criteria is more than 15.5%, the SMD will be confirmed	2. SD/SMD; then, ^18^F-FDG PET (SCAN 2) assessment is required after 3-4 weeks: ii) when the percent change in SULpeak is less than or equal to 15.5%, this pattern will be confirmed as PMD.	
	≥4 months of clinical benefit	≥6 months of clinical benefit	≥6 months of clinical benefit	No clinical benefit
PERCIMT Anwar et al., 2018 (Anwar et al., 2018)	Disappearance of metabolically active lesions	Disappearance of some metabolically active lesions	Neither CR/CMR, *p*R/PMR, nor *p*D/PMD	1.4 new lesions with functional size <1.0 cm	
				2.3 new lesions with functional size >1.0 cm	
				3.2 new lesions with functional size >1.5 cm	
iPERCIST Goldfarb et al., 2019 (Goldfarb et al., 2019)	Complete resolution of FDG uptake within the target lesion	1. ≥30% decrease in the target tumor FDG SUL_peak_or	Neither CR/CMR, *p*R/PMR, nor *p*D/PMD	1. ≥ 30% increase in FDG SUL_peak_ or advent of new FDG-avid lesions (UPMD)	
		2. confirmed as PMR by SCAN-3 from UPMD at SCAN-2		2. Need to be confirmed by a third PET (second posttreatment scan) at 4–8 weeks later (CPMD); if progression is followed by PMR or SMD, the bar is reset	
				(Clinical stability is considered when deciding whether treatment is continued after UPMD)	
	Responders	Responders	Responders	Non-responders	
imPERCIST5 Ito K et al., 2019 (Ito et al., 2019)	Complete resolution of FDG uptake within all lesions to a level of less than or equal to that of the mean liver activity and that is indistinguishable from the background (blood-pool uptake)	Reduction of at least 30% in the sum of SUL_peak_ of all target lesions detected at baseline and an absolute drop of 0.8 SUL_peak_ units	Neither CMR, PMR, nor PMD.	1. Increase in at least 30% in the sum of SUL_peak_ of all target lesions detected at baseline, and an absolute increase in 0.8 SUL_peak_ units with exclusion of infection/treatment effect	Target lesion: up to five measurable target lesions, typically the five hottest lesions among all lesions, including new lesions, and no more than two per organ
				Patients with appearance of new FDG-avid lesions must be confirmed 4–8 weeks later	
		PMR or SMD with appearance of new FDG-avid lesions at 4–8 weeks must be reevaluated	PMR or SMD with appearance of new FDG-avid lesions at 4–8 weeks must be reevaluated		

^18^F-FDG: 2-deoxy-2-[^18^F]-fluoro-D-glucose; PET/CT: positron emission tomography/computed tomography; CR: complete response; CMR: complete metabolic response; PR: partial response; PMR: partial metabolic response; SD: stable disease; SMD: stable metabolic disease; PD: progressive disease; PMD: progressive metabolic disease; SUL_peak_: peak standardized uptake values normalized by lean body mass; FDG: fluorodeoxyglucose; UPMD: unconfirmed progressive metabolic disease; CPMD: confirmed progressive metabolic disease; HPD: hyper-progression.

2) PET Response Evaluation Criteria for Immunotherapy (PERCIMT) (Anwar et al., 2018): PERCIMT, which demands two (pretherapeutic and posttherapeutic) PET/CT scans, suggests the number of newly emerged FDG-avid lesions on posttherapy PET/CT as cut-off value to identify the treatment failure in patients under immunotherapy which is quite different from the other criteria. Compared to other criteria, PERCIMT simplifies the assessment protocol by focusing on the limited number of new lesions. In the original study of PERCIMT, an absolute number of four new ^18^F-FDG-avid lesions, irrespective of their size, gave a reliable indication of nonresponse to treatment, and when the cut-off of newly emerged FDG-avid lesions with functional size < 1.0 cm was four, this assessing system showed the high sensitivity (84%) and specificity (100%). However, this research was also limited to the relatively small number of patients (N = 41), single cancer type (metastatic melanoma), and monotherapy (ipilimumab).

3) Immune PERCIST (iPERCIST) (Goldfarb et al., 2019): iPERCIST, which was adapted from PERCIST and the immune RECIST (iRECIST), demands two or three PET/CT scans (pretreatment, 2 months later after immunotherapy and 4–8 weeks after the second scan when necessary). iPERCIST’s dual-time-point evaluation is designed to differentiate the unconfirmed progressive metabolic disease (UPMD) from the confirmed progressive metabolic disease (CPMD). Then, the UPMD (PMD at SCAN-2) needs to be reevaluated 4–8 weeks later at SCAN-3. The continuation of treatment after the first evaluation by SCAN-2 was according to the physician’s judgment, metabolic response and the deterioration of clinical status, as described in iRECIST. If this UPMD (PMD at SCAN-2) is still considered as UPMD at SCAN-3, the UPMD at SCAN-3 is finally confirmed as CPMD. Patients with complete metabolic response (CMR), partial metabolic response (PMR), stable metabolic disease (SMD), or UPMD followed by PMR or SMD were classified as responders. Patients with UPMD associated with clinical deterioration or UPMD followed by CPMD were classified as non-responders. iPERCIST more effectively avoids the interference from pseudo-progression and HPD as the third scan could be administrated. The original clinical trial of iPERCIST showed the reliable ability to identification for responsiveness: responders continued treatment for a mean of 10.7 months (range 3.8–26.3), OS was longer for responders than that for non-responders (19.9 *vs*. 3.6 months, log-rank *P* = 0.0003), and the 1-year survival rates were 94% for responders and 11% for non-responders (Goldfarb et al., 2019). Nevertheless, this original trial was also developed through small sample research (N = 28), single cancer type (NSCLC), and monotherapy (nivolumab, a PD-1 blocker), and the prognostic value of iPERCIST should be confirmed in large prospective multicentric studies.

4) Immunotherapy-modified PERCIST5 (imPERCIST5) (Ito et al., 2019): imPERCIST5 requires several PET/CT scans, and especially when PMR or SMD is with newly emerged lesions, several scans should be administrated to reevaluate. The key difference of imPERCIST5 lies in the interpretation of new lesions on the posttreatment scan. In imPERCIST5, tumor response was assessed by the change in the sum of SUL_peak_ of up to five lesions. New lesions do not necessarily result in a scan to be classified as PMD, for the appearance of new lesions that resolved spontaneously and were probably inflammatory in nature. Thus, imPERCIST5 could efficiently discern real progression from pseudo-progression and irAEs. According to the original clinical analysis, imPERCIST remained prognostic (hazard ratio, 3.853; 95% confidence interval, 1.498–9.911; *P* = 0.005) (Ito et al., 2019). Of note, in the original study of imPERCIST5, whereas imPERCIST5 reduces overdiagnosis of progressive disease, new lesions in patients with PMR or SMD by imPERICST5 were eventually found to have metastases in 55% of the cases. Thus, if the patients have the decrease or no change in metabolism of existing target lesions as well as the appearance of new lesions, the prognosis appears indeterminate. Then biopsy should be considered before any change in treatment. imPERCIST5 was still produced based on a clinical study with only 60 metastatic melanoma patients treated with ipilimumab. A case study with assessment on PERCIMT and imPERCIST5 is provided in Figure 1.

These assessment criteria above discovered the limitations of current morphologic criteria, such as response without lesion volumetric changes and individual measurement differences among readers. Then, they all highlight the value of ^18^F-FDG PET/CT (e.g., the high repeatability and accuracy of measurement for semi-quantitative parameters among readers) in responsiveness evaluation and prognosis in malignancies with immunotherapy, and three of the four assessment systems suggested more than one posttreatment PET/CT scan. Furthermore, they proposed their own resolutions to overcome the limitations of anatomic criteria: PECRIT and iPERCIST focused on the overall metabolic level in the whole body; PERCIMT emphasized the number of the newly FDG-avid lesions; and imPERCIST5 stressed the limited target lesions’ FDG-uptake values. However, it must be admitted that these criteria are in diversity and just verified in small samples at present. Only one of them was developed from a study based on NSCLC patients. Thus, large-sample multi-center validation and further improvement are necessary in the future. Moreover, given the diversity of the metabolic level among NSCLC subtypes (glucose metabolism level of lung adenocarcinoma is lower than that of other NSCLC pathological subtypes (Lopci et al., 2016)), the dedicated evaluation criteria oriented to NSCLC or NSCLC subtypes should be developed. In addition, the increase in radiation dose and high cost of PET/CT scan also become the reasons restricting the application of assessment criteria based on ^18^F-FDG PET/CT. Above all, the lack of specificity of FDG is the main limitation for ^18^F-FDG PET/CT. It is reported that an increased consumption of glucose may be due either to lymphocytes’ activation, which reflects the adaptive host response to tumor and is associated with a more favorable outcome, or to neutrophils’ activation, promoting an inflammatory cascade leading to tumor progression and dismal prognosis (De Larco et al., 2004). Therefore, the posttherapeutic assessment is needed more than once to confirm the right status.

### 2.2 Specific PET Probes for Immunotherapy

New molecular probes arise that directly target the key molecules of immune checkpoint pathways and immune responses. They can not only image the PD-1/PDL-1/CTLA-4 status or lymphocyte infiltrations of tumor tissue and better understand the uptake and distribution of agents/molecules and their mechanisms, but also be useful to monitor patients initially presenting sensitivity to ICIs and then showing an acquired resistance after a variable period of time.

The novel PET probes and their research progression are presented in Table 4. Fundamental research demonstrated the high target specificity and fulfilling affinity and adequate tumor penetration of these probes. The tracers under clinical settings have authenticated the safety of humans. During the imaging experiments, the uptake of tracers has shown sufficient resolution, which can assess potential heterogeneity within each lesion and among lesions from the same patients. Furthermore, in some studies concerning the assessment of response to immunotherapy, significant correlations were displayed between the grade of uptake of tracers and the response. Beyond the scope of PET imaging, promising molecular structures can also be targeted using single-photon emission tomography ligands (Table 4) and provide another potential and feasible choice for response evaluation and prediction of prognosis in malignancies with immunotherapy. However, there are still limitations such as high hepatic and splenic uptake that affect the detection of abdominal lesions, long-circulating half-life, and radiation exposure for patients. In addition, most of them are still under the preclinical research phase, and only preliminary human studies have been carried out for some probes. Therefore, further research, improvement, and validation of specific probes to ICIs will continue in the future.

**TABLE 4:** Novel PET probes for immunotherapy.

Probe	Target	Study	PET probe	Experiment stage (subject)	Characteristics
Anti-PD-1 antibody	PD-1-expressing tumor-infiltrating lymphocyte	Liu et al., 2021 (Liu et al., 2021)	^68^Ga-NOTA-Nb109	Preclinical experiment (cell and animal models bearing different tumors)	^68^Ga-NOTA-Nb109 can specifically target endogenous PD-L1 and dynamic monitoring of the change of PD-L1 expression and could guide the immunotherapy and immunochemotherapy for refractory cancers
		Kelly et al., 2021 (Kelly et al., 2021)	^89^Zr-REGN3504	Preclinical experiment (mice and monkeys)	1.^89^Zr-REGN3504 specifically localized to spleen and lymph nodes in the PD-1/PD-L1 humanized mice
					2.^89^Zr-REGN3504 immuno-PET accurately detected a significant reduction in splenic PD-L1 positive cells following systemic treatment
		Niemeije et al., 2021 (Niemeijer et al., 2021)	^89^Z-pembrolizumab	Clinical experiment (NSCLC patients)	A significant correlation between the grade of uptake of the traces and the response assessed after 3 months of nivolumab was observed
		Li et al., 2020 (Li et al., 2021)	^89^Zr-N-sucDf-pembrolizumab	Preclinical experiment (healthy cynomolgus monkeys)	Preferential uptake in the lymphoid tissues, including the lymph nodes, spleen, and tonsils, was shown
		England et al., 2018 (England et al., 2018)	^89^Zr-DF nivolumab	Preclinical experiment	There was highly specific binding of ^89^Zr-DF nivolumab to activated T-cell infiltrating tumors in humanized murine models
		Cole et al., 2017 (Cole et al., 2017)	^89^Zr-nivolumab (BMS-936558)	Preclinical experiment (healthy non-human primates)	A study of biodistribution and clearance of BMS-936558 in animals
Anti-PD-L1 antibody	PD-L1-expressing tumor cell	Laffon et al., 2021 (Laffon and Marthan, 2021)	^18^F-BMS-986192	Clinical experiment (NSCLC patients)	A quantitative research: the ratio of SUV normalized for body weight to plasma concentration might be probed as a complementary possible simplified parameter, that is correlated with Ki/(kb + λ) within 50–55 min after injection
		Huisman et al., 2020 (Huisman et al., 2020)	^18^F-BMS-986192 (anti-PD-L1 adnectin)	Clinical experiment (NSCLC patients), quantitative research	SUV normalized for body weight at 60 min after injection may be a relevant simplified parameter to quantify tumor uptake for baseline PET studies
		Bridgwater et al., 2018 (Bridgwater et al., 2020)	^89^Zr-Df-F (ab')2	Preclinical experiment (Melanoma Mouse Mode)	PET/CT images clearly showed that ^89^Zr-Df-F (ab')2 possessed superior pharmacokinetics and imaging contrast over the radiolabeled full antibody, with much earlier and higher tumor uptake (5.5 times more at 2 h after injection) and much lower liver background (51% reduction at 2 h after injection)
		Truillet et al., 2018 (Truillet et al., 2018)	^89^Zr-C4	Preclinical experiment	1.^89^Zr-C4 can specifically detect antigen in human NSCLC and prostate cancer models endogenously expressing a broad range of PD-L1
					2.^89^Zr-C4 detects mouse PD-L1 expression changes in immunocompetent mice, suggesting that endogenous PD-1/2 will not confound human imaging
					3.^89^Zr-C4 could detect acute changes in tumor expression of PD-L1 due to standard of care chemotherapies
		Bensch et al., 2018 (Bensch et al., 2018)	^89^Zr-atezolizumab	Clinical experiment (bladder cancer, NSCLC, or TNBC patients)	1. Tumor uptake was generally high but heterogeneous, varying within and among lesions, patients, and tumor types. 2. Clinical responses in patients were better correlated with pretreatment PET signal than IHC- or ribonucleic acid-sequencing-based biomarkers
		Xing et al., 2019 (Xing et al., 2019)	^99m^Tc-NM-01	Clinical experiment (NSCLC patients)	1. Intra-tumoral and inter-tumoral heterogeneity was observed
					2. Primary tumor: blood-pool ratios at 2 h correlated with IHC.
Anti-CTLA-4 antibodies	CTLA-4-expressing activated T cells and some tumor cells	Ehlerding et al., 2019 (Ehlerding et al., 2019)	^64^Cu-NOTA-ipilimumab-F (ab')2	Preclinical experiment (mice)	PET imaging with both ^64^Cu-NOTA-ipilimumab and ^64^Cu-NOTA-ipilimumab-F (ab')2 was able to localize CTLA-4+ tissues
		Ehlerding et al., 2017 (Ehlerding et al., 2017)	^64^Cu-DOTA- ipilimumab	Preclinical experiment (mouse models of NSCLC)	^64^Cu-DOTA- ipilimumab can correctly localize the tumor, but a link was found with the receptor on the cell surface rather than in the intracellular domain
Anti-interferon-γ	Activated lymphocytes inside tumor lesions	Gibson et al., 2018 (Gibson et al., 2018)	^89^Zr-anti-IFN-γ	Preclinical experiment (mouse with mammary tumors)	The activation status of cytotoxic T cells is annotated by ^89^Zr-anti-IFN-γ PET, providing valuable non-invasive insight into the function of immune cells *in situ*
Protease granzyme B	Cytotoxic CD8^+^ T cells and natural killer cells	Larimer et al., 2017 (Larimer et al., 2017)	^68^Ga-NOTA-GZP	Preclinical experiment (human melanoma specimens)	Granzyme B PET imaging can serve as a quantitatively useful predictive biomarker for efficacious responses to cancer immunotherapy
Interleukin-2	Tumor/tissue infiltrating T lymphocytes	Markovic et al., 2018 (Markovic et al., 2018)	^99m^Tc-HYNIC-IL-2	Clinical experiment (melanoma patients with ipilimumab or pembrolizumab)	1. Safety and feasibility are verified
					2. Detect TIL and distinguish between true progression from HPD.

PET: positron emission tomography; PD-1: programmed cell death 1; PD-L1: programmed cell death ligand 1; NSCLC: non–small cell lung cancer; SUV: standardized uptake value; CT: computed tomography; PET/CT: positron emission tomography/computed tomography; TNBC: triple-negative breast cancer; IHC: immunohistochemistry; CTLA-4: cytotoxic T-lymphocyte associated-protein 4; IFN: interferon; IL-2: interleukin-2; TIL: tumor lymphocyte infiltration; HPD: hyper-progression.

## 3 Conclusion

Immunotherapy represents a powerful approach if used in selected oncological populations, including NSCLC patients. At present, PET/CT with ^18^F-FDG is an effective tool in identifying atypical patterns and adverse effects associated with immunotherapy, evaluating response, and predicting prognosis for NSCLC patients with ICIs, especially adopting semi-quantitative parameters and functional assessing criteria. Novel PET probes targeting key molecules relevant to ICIs and treatment response provide the promising perspective to more specifically screen oncological population that will benefit from immunotherapy and continuously assess ICI response in the future.
